# Alumni Day at the Harvard Dental School

**Published:** 1899-10

**Authors:** William H. Potter


### Domestic Correspondence.

### ALUMNI DAY AT THE HARVARD DENTAL SCHOOL.

To the Editor:

Sir,—For several years past it has been the custom for the alumni of the Harvard Dental School to assemble at their school building for the purpose of keeping alive their acquaintance one with another, for inspecting the work done by students of the school, and for listening to papers on current topics and observing practical operations. The day selected for this purpose is the Monday before the commencement day of Harvard University. This year "Alumni Day" was celebrated on June 26, and was an enjoyable and profitable occasion. In no way could a concise idea of what "Alumni Day" means be better given than by quoting the programme prepared for the occasion.

### Programme of Alumni Day, June 26, 1899.

*First Floor—Left.*

MECHANICAL LABORATORY.

Specimen Cases in Mechanical Dentistry.

(P. W. Moriarty, D.M.D., Demonstrator of Mechanical Dentistry.)

Second-year Class, in charge of Robert J. McMeekin, D.M.D., Assistant Demonstrator of Mechanical Dentistry.

Third-year Class, in charge of Thomas B. Hayden, D.M.D., Instructor in Mechanical Dentistry.

Specimen Cases in Porcelain Inlay, Continuous Gum, and Tooth Carving.

(Arthur H. Stoddard, D.M.D., Clinical Lecturer in Mechanical Dentistry.)

In charge of H. DeWitt Cross, D.M.D., Instructor in Mechanical Dentistry.

Specimen Cases in Crown- and Bridge-Work.

(William P. Cooke, D.M.D., Instructor in Crown- and Bridge-Work and in Metallurgy.)

In charge of Allen S. Burnham, D.M.D., Instructor in Mechanical Dentistry.

IMPRESSION-ROOM.

Cases of Fractured Jaw, Cleft Palate, and appliances used, patients being present.

In charge of P. W. Moriarty, D.M.D., Instructor in the Mechanical Treatment of Fractured Jaws and Cleft Palate.

### EXTRACTING-ROOM.

Photographs, illustrating methods of extracting.

In charge of Nathan P. Wyllie, D.M.D., Instructor in Materia Medica and Anæsthesia.

*Second Floor—Right.*

### EAST OPERATING-ROOM.

Practical Cases, patients being present, showing work done by Students in Operative Dentistry.

In charge of Joseph T. Paul, D.M.D., Demonstrator of Operative Dentistry.

Orthodontia.

(Eugene H. Smith, D.M.D., Professor of Mechanical Dentistry and Orthodontia.)

In charge of Harry W. Haley, D.M.D., Instructor in Mechanical Dentistry.

Crown- and Bridge-Work.

(William P. Cooke, D.M.D., Instructor.)

In charge of Arthur W. Eldred, D.M.D., Instructor in Mechanical Dentistry.

### DENTAL MUSEUM.

(Balcony of the East Operating-Room.)

Waldo E. Boardman, D.M.D., Curator.

### LIBRARY.

(North end of West Operating-Room.)

Waldo E. Boardman, D.M.D., Librarian.

*Second Floor.*

### OFFICE.

Emergency Corps Methods.

In charge of William H. Potter, D.M.D., Lecturer in Operative Dentistry.

Specimen Work of First-Year Students.

In charge of Henry L. Upham, D.M.D., Instructor in Operative Dentistry.

*Second Floor—Left.*

### WEST OPERATING-ROOM.

CLINICS.

"Treatment of Salivary Calculus and Pyorrhœa Alveolaris."

Edward C. Briggs, M.D., D.M.D., '78, Boston, Mass.
William P. Cooke, D.M.D., '81, Boston, Mass.
Frederick Bradley, D.M.D., '86, Newport, R. I.
Charles M. Keep, M.D., D.M.D., '90, Boston, Mass.

"Porcelain Inlay."

Arthur H. Stoddard, D.M.D., '87, Boston, Mass.
Hugh K. Hatfield, '99, Boston, Mass.
(Demonstrations with Jenkins's Outfit.)
Carl A. R. Samsioe, '99, Stockholm, Sweden.

"Porcelain Carving and Backing Teeth."

Robert T. Moffatt, D.M.D., '95, Boston, Mass.

"Method of Crowning."

Carl A. R. Samsioe, '99, Stockholm, Sweden.

"Extension Crown and a Suspension Bridge for Lower Molars."

Horatio C. Meriam, D.M.D., '74, Salem, Mass.

Apparatus for Testing Cement Fillings.

Virgil C. Pond, B.P., D.M.D., '80, Boston, Mass.

Practical Demonstrations of Phosphate Cements, with patients present.

Edward S. Niles, D.M.D., '79, Boston, Mass.

(*Notice photographs of all the classes on the walls of this room.*)

*First Floor.*

LECTURE-ROOM A—10.30 O'CLOCK.

*Frederick Bradley, D.M.D., Newport, R. I., President Alumni Association, will preside.*

Eugene H. Smith, D.M.D., Dean of Harvard Dental School, will address the meeting.

"Dental Legislation."

Frederick A. Stevenson, L.D.S., D.D.S., D.M.D., '88, Montreal, Canada.

Symposium, "Do the Modern Forms of Gold Produce More Permanent Fillings?"

Cecil P. Wilson, D.M.D., '72, Boston, Mass.
Frederick E. Banfield, D.M.D., '79, Boston, Mass.
Dwight M. Clapp, D.M.D., '82, Boston, Mass.

"Mechanical Tests for Cements."

Virgil C. Pond, B.P., D.M.D., '80, Boston, Mass.

"The Chemistry and Mixing Phosphate Fillings."

Edward S. Niles, D.M.D., '79, Boston, Mass.

WILLIAM H. POTTER, D.M.D.

